# Case Report: Challenges in immunotherapy for the elderly: a case of refractory ICI-induced AIHA and thrombocytopenia in advanced gastric cancer

**DOI:** 10.3389/fimmu.2025.1679817

**Published:** 2025-10-22

**Authors:** Mingxin Yu, Yanhong Ding, Linlin Ji, Yi Zhang, Kun Wu, Junying Zhang, Rongxuan Cao, Yang Liu, Yandong Bian, Xin Shen, Yuhui Nie, Yanfang Gao, Shuzhen Liu, Guohua Yu

**Affiliations:** ^1^ Weifang People’s Hospital, Shandong Second Medical University, Weifang, China; ^2^ Oncology Laboratory of the First Affiliated Hospital, Weifang People’s Hospital, Shandong Second Medical University, Weifang, China; ^3^ Thoracic Surgery of the First Affiliated Hospital, Weifang People’s Hospital, Shandong Second Medical University, Weifang, China; ^4^ Hematology of the First Affiliated Hospital, Weifang People’s Hospital, Shandong Second Medical University, Weifang, China

**Keywords:** immune checkpoint inhibitors (ICIs), immune-related adverse events (irAEs), autoimmune hemolytic anemia (AIHA), thrombocytopenia, advanced gastric cancer

## Abstract

Immune checkpoint inhibitors (ICIs) have revolutionized cancer immunotherapy. However, immune-related adverse events (irAEs), particularly hematologic toxicities, remain rare but potentially life-threatening complications. Among these, autoimmune hemolytic anemia (AIHA) induced by ICIs is a clinically significant yet underrecognized condition. We report a rare case of ICI-induced AIHA concomitant with thrombocytopenia in an elderly female patient with advanced gastric cancer who had undergone immune monotherapy.

**Case description:**

An 84-year-old female with unresectable, PD-L1–high (combined positive score [CPS] = 55) advanced gastric adenocarcinoma developed a rare case of recurrent immune checkpoint inhibitor associated autoimmune hemolytic anemia and thrombocytopenia after sequential treatment with three ICIs: sintilimab (anti–PD-1), cadonilimab (a bispecific PD-1/CTLA-4 antibody), and ivonescimab (a bispecific PD-1/VEGF antibody). Initially, sintilimab monotherapy induced partial tumor regression; however, the patient later developed transfusion-dependent anemia and severe thrombocytopenia. Bone marrow aspiration revealed erythroid aplasia and characteristic teardrop poikilocytes, suggesting marrow stress or fibrosis. Reintroduction of ICIs triggered recurrent hematologic toxicity, pointing to a class-wide immune-mediated mechanism. Despite aggressive treatment with glucocorticoids, red blood cell and platelet transfusions, and comprehensive supportive care, the cytopenias persisted, and imaging confirmed disease progression. Ultimately, immune-related hematologic toxicity led to multiorgan failure and the patient’s death.

**Conclusion:**

This case underscores the diagnostic complexity and therapeutic challenges associated with immune checkpoint inhibitor induced autoimmune hemolytic anemia in elderly patients, particularly those experiencing immunosenescence and possessing limited hematopoietic reserve. The mechanisms underlying this phenomenon remain poorly understood, highlighting the urgent need for mechanistic investigations, individualized immunotherapeutic strategies, and vigilant hematologic monitoring in vulnerable populations.

## Introduction

Immune checkpoint inhibitors have revolutionized cancer treatment by enhancing antitumor immunity. Unlike traditional chemotherapy, ICIs are associated with fewer off-target side effects (1). Immune checkpoint inhibitors activate the body’s immune response, primarily by blocking the PD-1/PD-L1 and CTLA-4 pathways. This enables T cells to identify and attack tumor cells using the body’s own defenses. As a result, immunotherapy has emerged as a promising approach for achieving long-lasting disease control, and in some cases, even potential cures for certain cancers (2).

However, the widespread clinical adoption of immune checkpoint inhibitors has been accompanied by a growing incidence of immune-related adverse events (irAEs), the severity of which ranges from mild to life-threatening. These toxicities span multiple organ systems, including dermatologic manifestations such as rash and hyperpigmentation, gastrointestinal complications such as immune-mediated enteritis, endocrine dysfunction exemplified by thyroid abnormalities, and cardiopulmonary events such as immune-related pneumonitis and myocarditis, in severe cases, hematologic complications may also occur. Although hematologic toxicities are relatively uncommon, they present a significant risk of morbidity and mortality, necessitating careful clinical monitoring. Among these, autoimmune hemolytic anemia is a severe and potentially fatal complication. A meta-analysis of 9,324 patients receiving ICI therapy reported incidence rates of anemia, neutropenia, and thrombocytopenia as 9.8%, 0.94%, and 2.8%, respectively (3). According to the U.S. Food and Drug Administration (FDA) Adverse Event Reporting System, AIHA represents approximately 0.06% to 0.25% of all ICI-associated adverse event reports, with PD-1/PD-L1 inhibitors being more frequently implicated than CTLA-4 antagonists.

The REISAMIC registry, a prospective multicenter cohort study monitoring patients undergoing anti–PD-1 or anti–PD-L1 therapy, identified four cases of hematologic immune-related adverse events among 745 enrolled participants. In a broader analysis involving 5,923 patients from 19 clinical trials, the overall incidence of immune-related adverse events was reported as 3.6%, with 0.6% of patients experiencing autoimmune hemolytic anemia and 0.3% experiencing pancytopenia or pure red cell aplasia (4). Despite accumulating clinical experience with ICIs, the epidemiology of ICI-associated AIHA (ICI-AIHA) remains poorly understood. However, emerging evidence suggests a significantly higher risk in individuals with underlying autoimmune diseases or pre-existing hematologic conditions (5).

Clinically, ICI-AIHA often presents with subtle and diverse features, making early diagnosis challenging. It may be mistakenly attributed to cancer progression or chemotherapy-induced anemia, which can delay appropriate treatment. While initial treatment with corticosteroids and immunosuppressants can induce remission in some patients, 30% to 40% of patients require second-line therapies such as rituximab or complement inhibitors. Furthermore, discontinuing immunotherapy—often due to severe irAEs—poses a dilemma, as it may compromise cancer control and increase the risk of tumor relapse (6).

## Case presentation

An 84-year-old female patient presented with persistent abdominal distension of unknown etiology and was admitted to the Department of Gastrointestinal Surgery for further evaluation. Upper gastrointestinal endoscopy revealed a mass in the gastric antrum, accompanied by pyloric obstruction. Biopsy results confirmed moderately to poorly differentiated adenocarcinoma of the gastric antrum. The patient then underwent diagnostic laparoscopy and a palliative gastrojejunostomy at Shandong Provincial Hospital under general anesthesia. Intraoperative findings included a 6 × 5 cm firm mass in the antrum causing pyloric obstruction, with serosal invasion extending into the transverse mesocolon, pancreatic head, and descending duodenum. Clustered lymphadenopathy was noted at the hepatic hilum, and the tumor was deemed unresectable due to fixation.

Postoperative immunohistochemistry revealed a PD-L1 combined positive score (CPS) of 55, along with CerbB-2 (0), MLH1 (−), MSH2 (+), MSH6 (+), and PMS2 (−), indicating partial mismatch repair deficiency. Clinical staging was identified as cT_4b_N_+_M_0_, consistent with stage III disease. Based on the Chinese Society of Clinical Oncology (CSCO) guidelines for gastric cancer and supporting evidence, monotherapy with S-1 (Tegafur–Gimeracil–Oteracil) combined with immunotherapy was recommended. Following a shared decision-making process, the patient chose immune monotherapy with Sintilimab (200 mg/day), which was administered on May 12, 2023.

**Figure 1:**
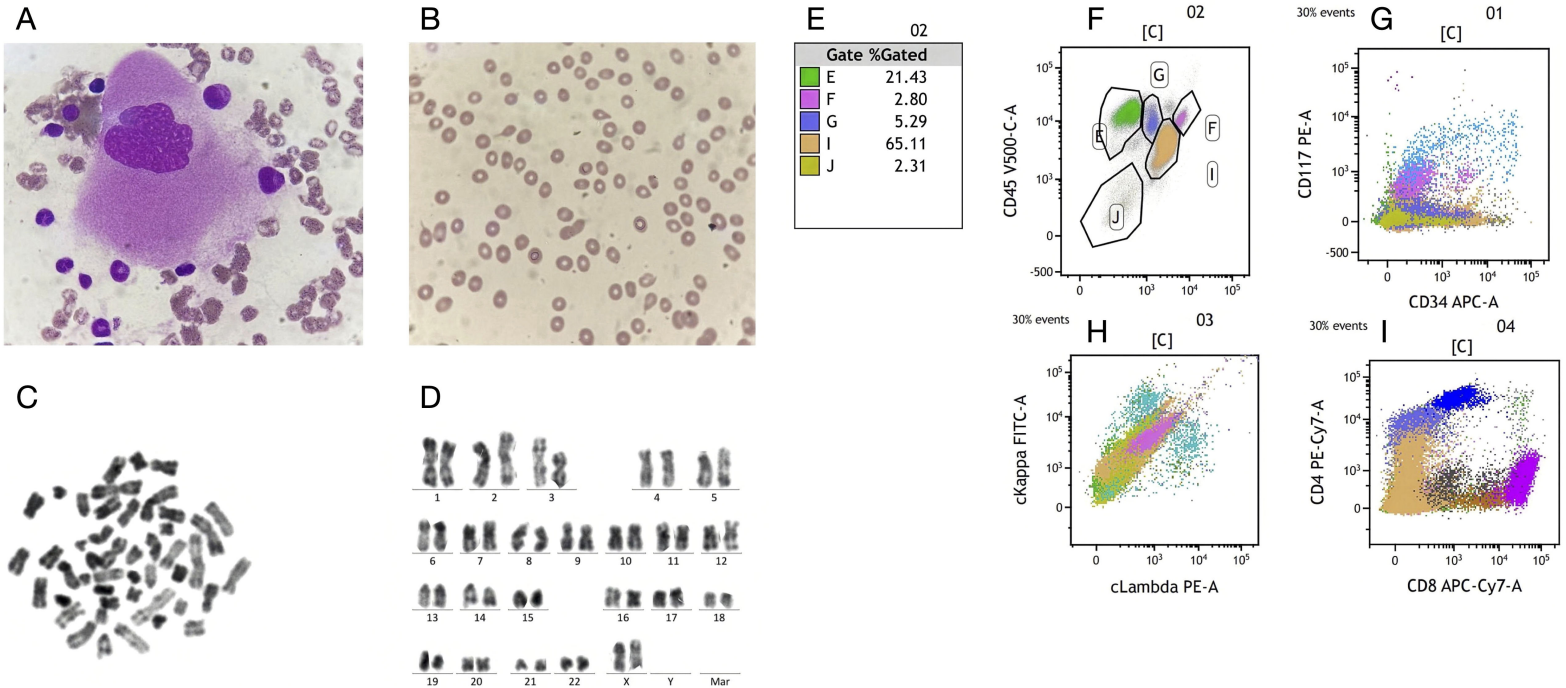
Integrated findings from bone marrow aspirate analysis. **(A, B)** Blood smear results indicated an increased percentage of granulopoiesis, absence of the red blood cell lineage, visible megakaryocytes, tear-drop shaped red blood cells in the peripheral blood, and thrombocytopenia.**(C, D)** Karyotypic analysis of the patient showed no chromosomal abnormalities. **(E–I)** Flow cytometric profiling revealed a physiologically balanced distribution of immune cell subsets, with no evidence of aberrant immunophenotypes or clonal expansion, consistent with an intact immune architecture and hematopoietic homeostasis.

Prior to initiating immunotherapy on May 13, 2023, the patient underwent a complete blood count at Weifang Municipal Hospital of Traditional Chinese Medicine on May 1, 2023, which revealed hemoglobin (HGB) of 99 g/L and platelet (PLT) count of 194 × 10^9^/L. The baseline hemoglobin level was below the normal range, a finding that may be attributable to the patient’s advanced age, as age-related decline in bone marrow hematopoietic reserve is well recognized. Following a one-week treatment course with sintilimab, subsequent laboratory investigations revealed a pronounced decline in both hemoglobin concentration and platelet count. By early June 2024 (approximately two weeks after initiation of therapy), hemoglobin levels had precipitously fallen to 53 g/L, accompanied by a concomitant decrease in platelet count to 20 × 10^9^/L, accompanied by severe fatigue. The patient was transferred to the hematology department for further evaluation and management of suspected hematologic toxicity. To investigate the cause of severe anemia, a bone marrow aspiration and peripheral blood smear were performed on June 8, 2023. The bone marrow smear showed an increased proportion of lymphocytes and granulocytes, with a notable absence of erythroid precursors. Megakaryocytes were rare, with only three observed (**Figure 1A**), and thrombocytopenia was noted. The peripheral blood smear showed anisocytosis with frequent tear-shaped red blood cells and a significant number of large granular lymphocytes (**Figure 1B**), comprising 42% of the total lymphocyte count, 2% of which were atypical lymphocytes. Although the white blood cell count was within normal limits, platelet counts were significantly decreased, and some granulocytes exhibited excessive cytoplasmic granulation. Although no spherocytes were identified on the peripheral blood smear, serologic evidence for warm autoantibody–mediated autoimmune hemolytic anemia (wAIHA) has been established by a positive direct antiglobulin (Coombs) test performed at our center together with laboratory evidence of hemolysis. Given the pathophysiology of spherocyte formation—partial splenic phagocytosis of IgG-opsonized erythrocytes—the absence of spherocytosis likely reflects early-stage disease with mild hemolysis and/or pre-analytical and analytical factors (e.g., suboptimal selection of fields within the smear’s monolayer), which can obscure characteristic morphologic changes (7). Based on the evidence summarized by Barcellini, the premise that “early-stage disease corresponds to only mild hemolysis” is inconsistent with the established pathophysiology and clinical heterogeneity of autoimmune hemolytic anemia (AIHA). Disease severity in AIHA is determined not only by the pathogenic potential of autoantibodies but also by the efficiency of bone marrow erythroblastic compensation. Importantly, reticulocytopenia is not uncommon—particularly in severe or pediatric cases—and may result from autoimmune targeting of bone-marrow erythroid precursors. This mechanism provides a pathophysiological rationale for the occurrence of profound anemia despite apparently modest hemolytic indices in the peripheral blood, a scenario consistent with the bone-marrow finding of absent erythroid precursors in the present case (8). Immunophenotyping of the bone marrow and peripheral blood showed no abnormal expression in the myeloid, lymphoid, or erythroid cell lines. Chromosomal analysis revealed a normal karyotype (46, XX) with no structural or numerical abnormalities (**Figures 1C, D**). Flow cytometry did not suggest any immune phenotypic abnormalities indicative of hematologic malignancy (**Figures 1E–I**). On June 10, 2023, the patient underwent a direct antiglobulin (Coombs) test at our institution, which yielded positive results for IgG (2+) and negative results for anti-C3d. Although a drug-dependent antibody elution test was not performed, the patient’s medical history, corroborated by family inquiry, indicated no chronic comorbidities and no concomitant medication use during the course of immunotherapy. Thus, alternative drug-induced hemolytic anemia could be reasonably excluded. Hemolysis-related laboratory parameters obtained on June 6, 2023, demonstrated indirect bilirubin (IBIL) of 87 μmol/L, lactate dehydrogenase (LDH) of 487 U/L, and reticulocyte (RET) count of 4.5%. At our center, platelet antibody screening on June 9, 2023 yielded a weakly positive result for platelet-reactive antibodies. On July 11, 2024, platelet aggregometry using arachidonic acid and adenosine diphosphate as agonists (PAgT-AA+ADP) demonstrated impaired platelet aggregation. The patient had a lean body habitus with suboptimal nutritional status (BMI 17.9 kg/m²), serum albumin consistently around 40 g/L, and no notable abnormalities in serum electrolytes or hepatic and renal function tests. Importantly, no clinical evidence of active bleeding was observed during treatment.

**Figure 2:**
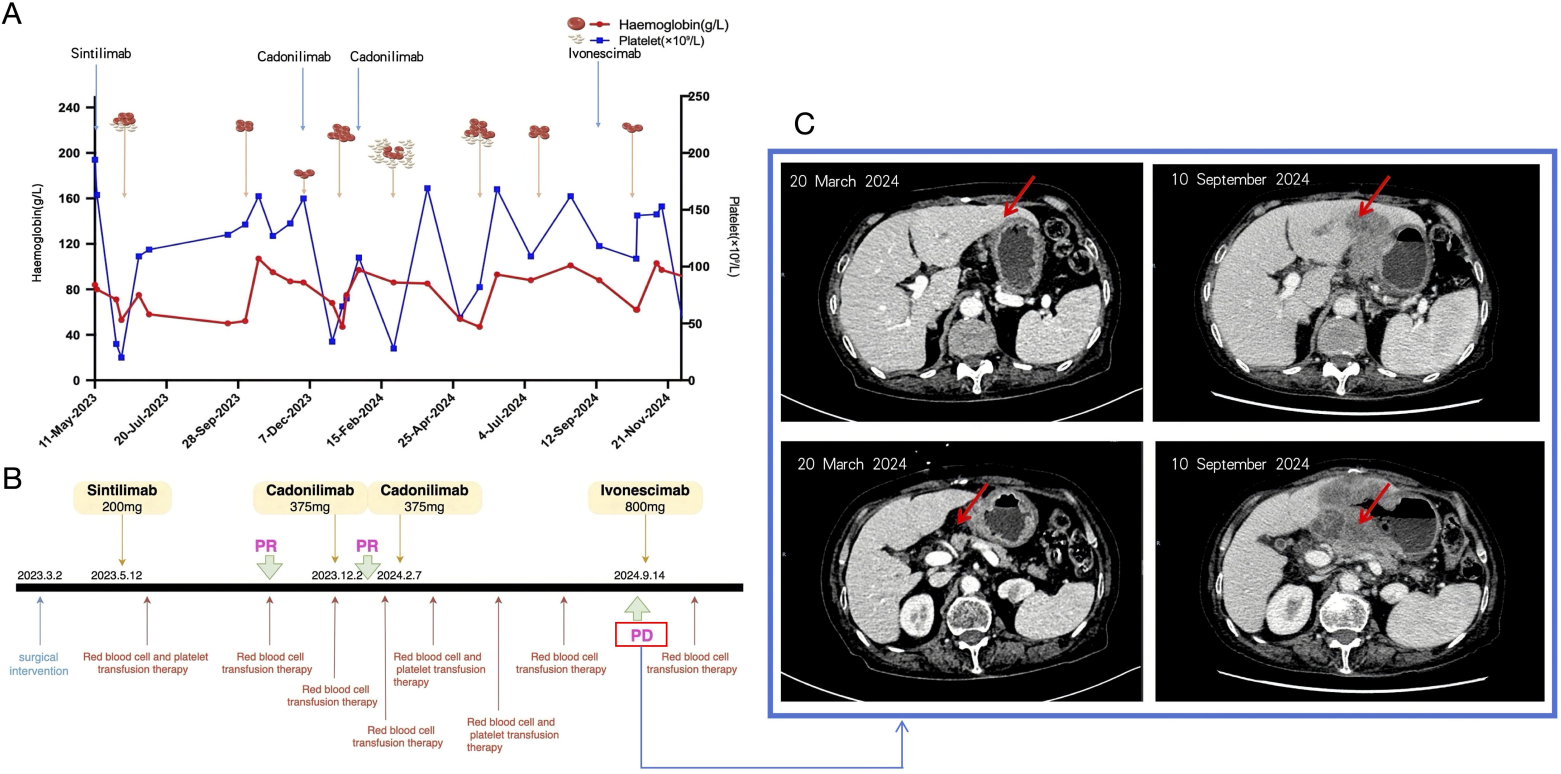
The process by which this patient was treated. **(A)**Trajectory of platelet counts and hemoglobin levels during the course of treatment. **(B)** Timeline of major therapeutic interventions administered to the patient. **(C)** The patient’s follow-up CT after 2 cycles of treatment with cardunculizumab showed metastases in the left lobe of the liver and enlarged abdominal and retroperitoneal lymph nodes, which were significantly more advanced than before.

Following a multidisciplinary evaluation by the hematology team, these findings—particularly the reduction in erythropoiesis, the presence of tear-drop shaped red blood cells, and persistent anemia requiring transfusions—were interpreted as secondary autoimmune hemolytic anemia, likely induced by immune checkpoint inhibitors in the context of advanced gastric adenocarcinoma. In the absence of evidence for clonal hematologic disease and no chromosomal, genetic, or molecular abnormalities, the hematology team suspected an ICI-related cause. Continuous monitoring was recommended, as no signs of primary hematologic disease were identified. Despite multiple transfusions, the patient’s hemoglobin levels showed only transient improvements. Platelet counts improved post-transfusion, but anemia persisted. Subsequent interventions included red blood cell transfusions, corticosteroid therapy, and supportive care, leading to a gradual increase in hemoglobin levels (**Figure 2**). The patient experienced recurrent hematologic toxicities during sequential immune checkpoint inhibitor (ICI) therapy. Upon initiation of sintilimab on May 11, 2023 (baseline hemoglobin, 99 g/L), hemoglobin precipitously declined within two weeks to <60 g/L (CTCAE v5.0 Grade 3 anemia) and platelets to ~20 × 10^9^/L (Grade 4 thrombocytopenia), necessitating transfusion support. Following cadonilimab administration on December 2, 2023, and February 7, 2024, hemoglobin frequently remained within Grade 2–4 ranges (nadir, 47 g/L), while platelets repeatedly dropped to Grade 3 (nadir, 28 × 10^9^/L). After initiation of ivonescimab on September 14, 2024, blood counts transiently improved but subsequently deteriorated in cycles, on December 11, 2024, laboratory tests revealed hemoglobin of 90 g/L (Grade 2 anemia) and platelets of 24 × 10^9^/L (Grade 4 thrombocytopenia). Supportive interventions included glucocorticoids, recombinant human thrombopoietin, traditional Chinese medicine, and transfusional therapy, but these yielded limited benefit. Given the patient’s profound cytopenia and heightened risk of infection, together with the partial reversibility of hematologic abnormalities under supportive care and the clinical priority of preserving antitumor immunity, no additional immunosuppressive agents were introduced. Despite recurrent Grade 3–4 hematologic irAEs of similar mechanism, ICIs were reintroduced following hematologic recovery, as previous administrations had achieved meaningful antitumor responses (partial response in October 2023 and January 2024, stable disease in March–April 2024) and the patient’s poor performance status precluded alternative therapeutic options. This course underscores a pragmatic, risk-adapted decision to balance sustained oncologic benefit with the management of life-threatening hematologic toxicities under close surveillance.

During treatment with multiple immune checkpoint inhibitors (sintilimab, cadonilimab, and ivonescimab), the patient developed drug-related secondary autoimmune hemolytic anemia and thrombocytopenia. Hemoglobin levels declined precipitously to a nadir of 62 g/L, despite transient increases following transfusions, and platelet counts decreased from 194 × 10^9^/L to as low as 31–32 × 10^9^/L. Notably, after a single infusion of cadonilimab (10 mg/kg on December 2, 2023), an acute 78% reduction in platelets was observed. Although interim imaging in January 2024 showed partial regression of abdominal and retroperitoneal lymphadenopathy, subsequent contrast-enhanced CT on September 10, 2024 revealed disease progression with increased gastric tumor burden, new hepatic metastases, and further lymph node enlargement. Clinically, the patient experienced worsening anemia, anorexia, nausea, and severe fatigue, necessitating repeated transfusions and symptomatic management.

Against the backdrop of a compromised baseline condition, severe immune-related adverse events (irAEs) accelerated tumor progression and precipitated rapid systemic decompensation. The subsequent development of infection, together with profound anemia and thrombocytopenia, further compounded clinical deterioration. Despite intensive supportive care, the patient’s condition continued to worsen, culminating in multi-organ failure and death on November 12, 2024.

**Figure 3:**
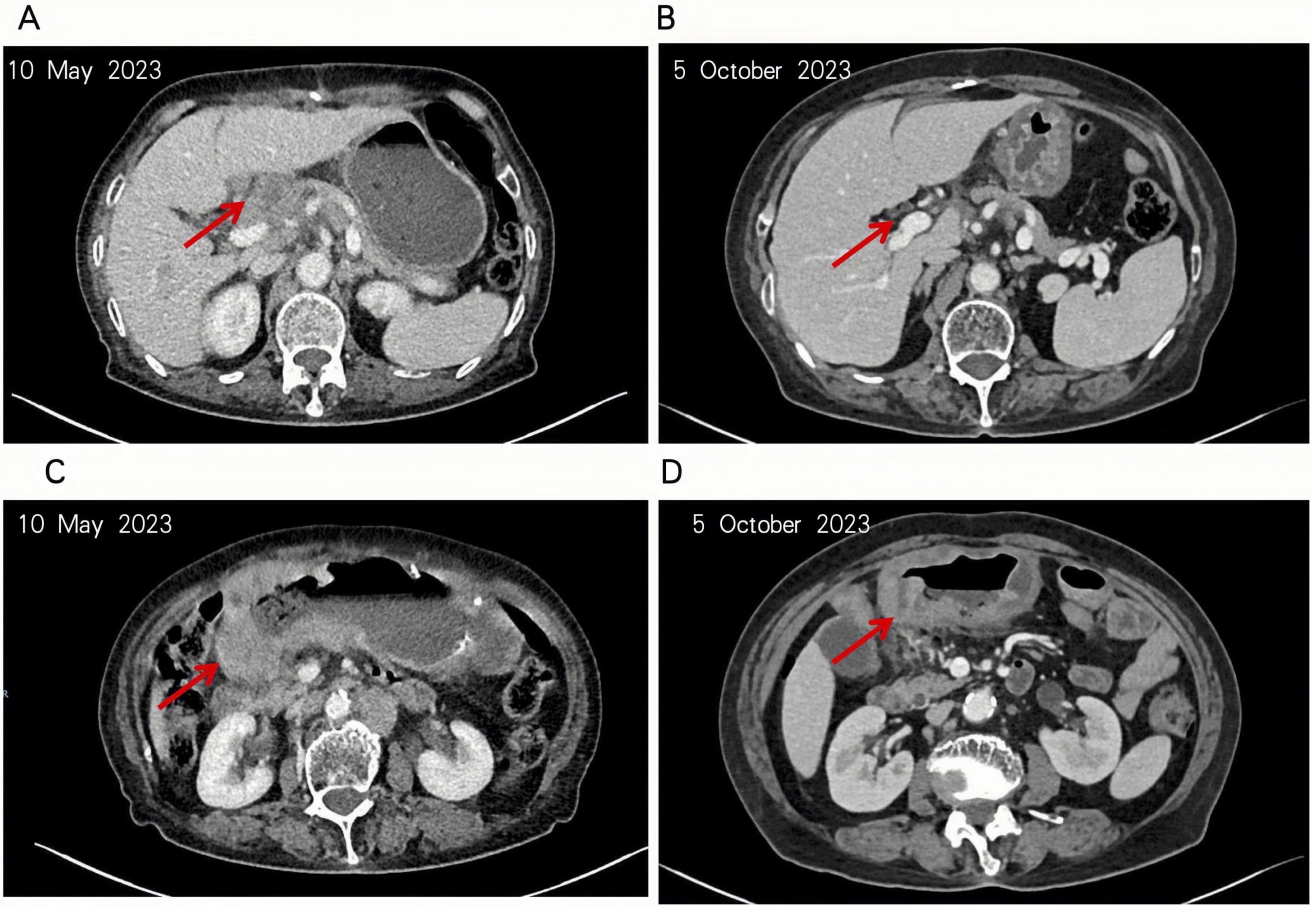
The patient’s tumor and hepatogastric interstitial lymph nodes were significantly reduced. **(A, B)** The patient’s lymph nodes in the hepatoportal and hepatogastric hiatus were significantly reduced as seen in the intravenous phase of enhanced CT. **(C, D)** Notable regression of the patient’s tumor in the gastric antrum observed in the same enhanced CT sequence.

The patient initiated sintilimab on May 12, 2023, but treatment was complicated by severe declines in hemoglobin and platelet counts, leading to discontinuation and irregular continuation of immunotherapy. The first efficacy evaluation was conducted after completion of a single treatment cycle, approximately 4.5 months later. Cross-sectional imaging on October 5, 2023, revealed substantial regression of both the primary tumor and regional lymph nodes (**Figure 3**), consistent with a partial response (PR). However, disease progression was documented on November 28, 2023, and therapy was switched to cadonilimab. Imaging on January 26, 2024, again showed PR, followed by stable disease on March 20 and April 3, 2024. Nevertheless, by September 10, 2024, radiographic assessment confirmed progression, prompting initiation of ivonescimab therapy.

## Discussion

Immune checkpoint inhibitors represent a transformative class of anticancer agents that exert their therapeutic effect by selectively targeting immunosuppressive pathways. By abrogating inhibitory signaling on T lymphocytes—particularly via blockade of PD-1/PD-L1 or CTLA-4 interactions—ICIs potentiate cytotoxic immune responses, thereby enhancing lymphocyte-mediated tumor eradication and markedly improving clinical outcomes in patients with advanced malignancies. However, despite their profound efficacy, ICIs may disrupt peripheral immune tolerance, giving rise to a spectrum of immune-related adverse events that can involve virtually any organ system and range from mild to life-threatening in severity.

In this case, an 84-year-old patient with advanced gastric carcinoma developed recurrent episodes of autoimmune hemolytic anemia following sequential administration of three mechanistically distinct immune checkpoint inhibitors: sintilimab, cadonilimab, and ivonescimab. Each ICI exposure was temporally associated with either new-onset or exacerbated hematologic toxicity, suggesting a causal relationship. Laboratory findings were consistent with active hemolysis, including abrupt declines in hemoglobin and platelet counts, transfusion dependence, and elevated lactate dehydrogenase (LDH) levels. The cyclical nature of hemoglobin decline and its only transient response to transfusion strongly indicated immune-mediated hemolysis. Taken together, the clinical course and laboratory evidence were highly suggestive of ICI-induced AIHA. This case underscores that, although ICIs have collectively reshaped cancer immunotherapy by overcoming immune tolerance, they may also exert divergent immunotoxic effects depending on their specific molecular targets and mechanisms of action.

Mechanistically, immune checkpoint inhibitors exert their antitumor effects by disrupting inhibitory pathways—most notably through blockade of PD-1, PD-L1, and CTLA-4—thereby releasing the brakes on T cell activity and amplifying antitumor immunity. However, this immune reactivation can become dysregulated, leading to aberrant T cell responses that target healthy tissues. Such immune overactivation may provoke hematologic immune-related adverse events, including autoimmune hemolytic anemia, immune thrombocytopenia (ITP), and, in rare cases, Evans syndrome—collectively referred to as immune-mediated cytopenias (9).

This pathological process is orchestrated by a complex network of immune cell interactions, wherein antigen-presenting cells such as dendritic cells activate autoreactive T lymphocytes, which in turn promote B cell differentiation into antibody-secreting plasma cells. These plasma cells produce pathogenic autoantibodies directed against hematologic targets, including erythrocytes and platelets (10).

Distinct immune checkpoint inhibitors elicit divergent immunopathogenic mechanisms. Sintilimab primarily enhances peripheral CD8^+^Tcell activity, thereby promoting B cell differentiation into plasma cells that secrete IgG-class warm autoantibodies. These autoantibodies mediate extravascular hemolysis via opsonization and subsequent phagocytosis of erythrocytes by splenic macrophages. By contrast, cadonilimab, through dual blockade of PD-1 and CTLA-4, not only augments the effector function of peripheral T cells but also enhances the priming of naive T cells within lymph nodes. Such amplified immune activation, driven by convergent immunopathological processes, may ultimately precipitate the development of Evans syndrome (11). In contrast, cadonilimab’s dual blockade of PD-1 and CTLA-4 not only augments the effector function of peripheral T cells but also potentiates the priming of naive T cells within lymph nodes. This intensified immune activation may elicit polyclonal B-cell responses and induce the production of IgM-type cold agglutinins, potentially triggering complement-mediated intravascular hemolysis. Such immunopathological processes may culminate in the development of Evans syndrome, characterized by concurrent autoimmune hemolytic anemia and immune thrombocytopenia (12).

**Figure 4:**
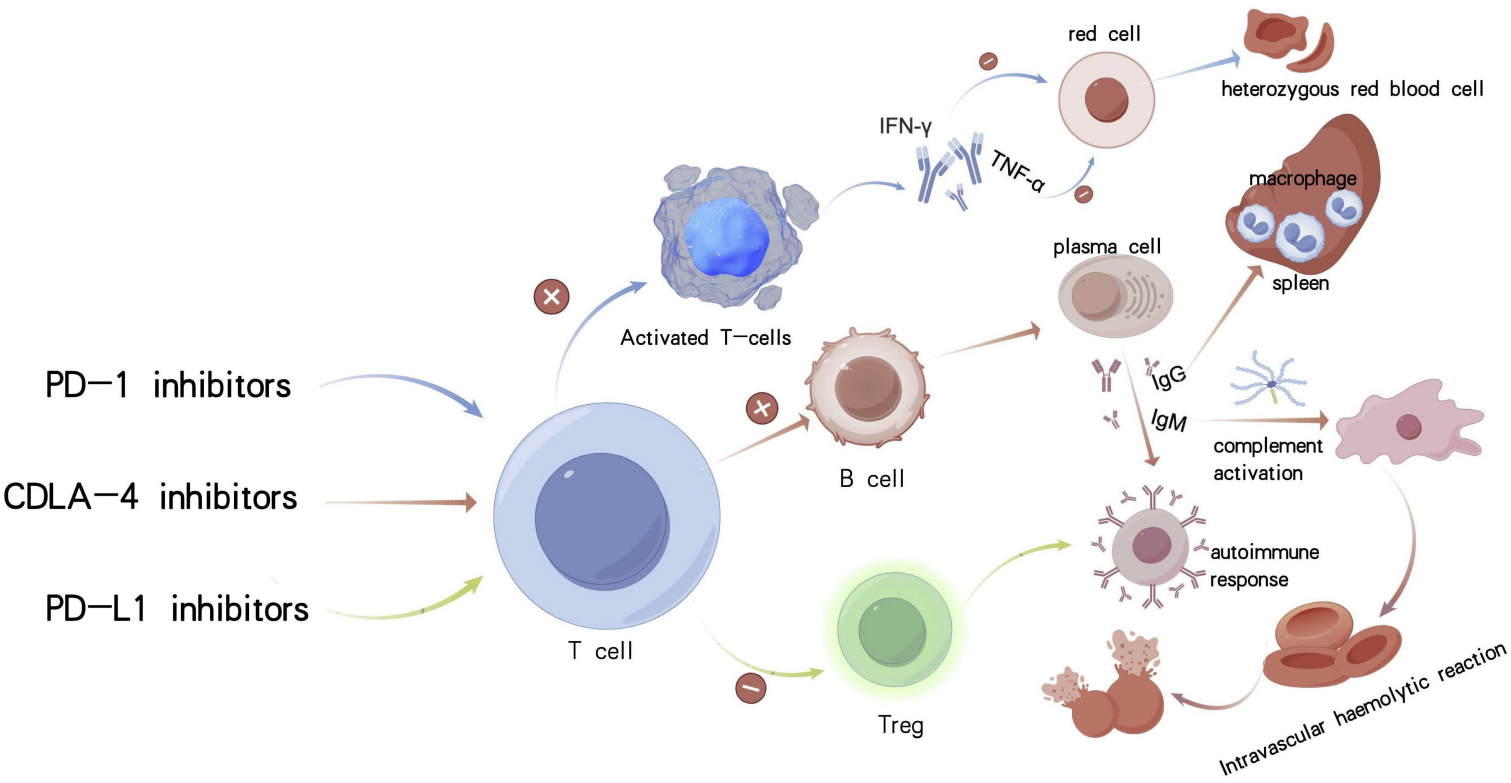
Mechanisms of autoimmune hemolytic anemia caused by three different types of immunological drugs.

Ivonescimab indirectly suppresses the activity of regulatory T cells (Tregs) by targeting the PD-L1 pathway, thereby modestly enhancing autoimmune responses while exhibiting relatively low hematologic toxicity (13). Among the various immune checkpoint inhibitors, bispecific antibodies, due to their dual immune-activating mechanisms, are associated with the highest hematologic toxicity (14), whereas PD-L1 inhibitors generally demonstrate a more favorable safety profile (**Figure 4**). Mechanistically, Fc receptor–dependent phagocytosis by macrophages plays a direct role in the clearance of blood cells. These autoimmune phenomena may occur idiopathically or as secondary features of immune dysregulation syndromes and malignancies, underscoring the critical role of immune tolerance breakdown in their pathogenesis (15). B cells are critically implicated in the pathogenesis of autoimmune hemolytic anemia. Emerging evidence suggests that immune checkpoint inhibitor-associated AIHA (ICI-AIHA) primarily arises from the disruption of peripheral immune tolerance, with particular involvement of regulatory T cell (Treg) dysfunction. Programmed cell death protein 1 (PD-1) inhibitors may exacerbate autoimmunity by impairing Treg-mediated suppression, thereby facilitating the activation of autoreactive B cells and promoting the production of red blood cell-specific autoantibodies, including immunoglobulin G (IgG). Notably, clonal expansion of T cells is observed in approximately 48.5% of patients with autoimmune hemolytic anemia, which may further disrupt B cell function (16). Cadonilimab, a bispecific antibody targeting both PD-1 and CTLA-4, may potentiate B cell co-stimulation via CTLA-4 modulation, thereby exacerbating complement-dependent hemolytic activity.

Recent investigations into the pathogenesis of autoimmune hemolytic anemia have revealed compelling evidence implicating complement activation as a critical driver of hemolysis. Utilizing standard hematological assays, phagocytosis-based functional tests, and flow cytometric analysis, researchers have demonstrated that extracellular vesicles derived from patient plasma are frequently coated with immunoglobulin isotypes IgA, IgG, and IgM, accompanied by marked complement component deposition. These findings suggest that complement-mediated mechanisms may represent an underrecognized but pivotal pathway contributing to erythrocyte destruction in AIHA (17). Moreover, the anti-angiogenic activity of PD-1/VEGF bispecific antibodies may further compromise the integrity of the bone marrow microenvironment, which, in concert with PD-1 pathway–mediated immune toxicity, exacerbates hematologic adverse events (18).

Advanced age is a critical and often underrecognized predisposing factor for ICI-associated autoimmune hemolytic anemia. Age-related biological processes, such as cellular senescence, are known to reshape immune functionality and may profoundly alter host responses to immune checkpoint inhibitors. In elderly individuals—such as the present case involving an 84-year-old patient—hallmarks of immunosenescence, including diminished regulatory T cell (Treg) function, clonal hematopoiesis, and a pro-inflammatory milieu, create a permissive environment for the development of ICI-induced AIHA. Senescent T cells are capable of secreting high levels of pro-inflammatory cytokines such as interleukin-6 (IL-6) and tumor necrosis factor-alpha (TNF-α), which in turn may facilitate the production of autoantibodies (**Figure 4**). Furthermore, the patient’s tumor exhibited high PD-L1 expression (combined positive score [CPS] = 55), which may have intensified immune activation and disrupted peripheral immune tolerance, thereby compounding hematologic toxicity (19).

Despite corticosteroids being the most frequently employed and widely considered the first-line therapy, their overall efficacy remains unsatisfactory. Current standard management includes vigilant monitoring, administration of hematopoietic growth factors, prophylactic antimicrobial measures, and transfusion of red blood cells or platelets when clinically indicated (20). Nevertheless, their overall efficacy in such cases remains unsatisfactory. However, these interventions are frequently accompanied by significant drawbacks, including bone pain, heightened risk of infection, limited therapeutic duration, and substantial financial burden. In elderly patients, those exhibiting high PD-L1 expression, or individuals predisposed to immune dysregulation, immunotherapeutic regimens should be selected with heightened caution. In such contexts, monotherapy with PD-L1 inhibitors may be preferable, alongside vigilant hematologic monitoring to promptly detect and manage treatment-related cytotoxicity (21).

Circadian rhythm disruption has emerged as a critical factor contributing to tumorigenesis and immune dysregulation. Leveraging a circadian rhythm disruption (CRD) score, researchers have developed prognostic models revealing that circadian rhythm–related genes (CRRGs) are intimately linked with multiple immune and metabolic pathways. Within these models, elevated CRD scores are strongly associated with attenuated responses to immunotherapy (22). These findings underscore the potential of optimizing chronotherapy—the alignment of immune checkpoint inhibitor administration with circadian rhythms—as a strategy to reduce the incidence of immune-related adverse events, such as ICI-induced autoimmune hemolytic anemia, while improving therapeutic outcomes. Moving forward, rigorously designed time-stratified clinical trials are warranted to determine whether circadian-informed ICI dosing schedules can mitigate toxicity without compromising, or possibly even enhancing, antitumor efficacy.

## Conclusion

We report a rare case of an 84-year-old female patient with advanced gastric cancer who developed recurrent immune-related hematologic toxicities—including immune checkpoint inhibitor-induced autoimmune hemolytic anemia and thrombocytopenia—following sequential treatment with three immune checkpoint inhibitors targeting distinct pathways: the PD-1 inhibitor sintilimab, the PD-1/CTLA-4 bispecific antibody cadonilimab, and the PD-L1 inhibitor ivonescimab. The patient experienced severe, treatment-refractory hemolysis during each therapeutic course, manifesting as profound anemia, erythroid dysplasia, transfusion dependency, and bone marrow suppression. This case highlights the mechanistic complexity of ICI-AIHA, with discussion centered on immune tolerance disruption, antibody-mediated hemolysis, immunosenescence, and chronotherapeutic considerations. Notably, the synergy between impaired immune tolerance in the elderly and elevated PD-L1 expression may have predisposed the patient to heightened hematologic immune toxicity. This report underscores the critical need for vigilant hematologic monitoring in elderly patients receiving immunotherapy and advocates for individualized therapeutic strategies tailored to host immune status.

## Data Availability

The raw data supporting the conclusions of this article will be made available by the authors, without undue reservation.

## References

[1] Hussaini S Chehade R Boldt RG Raphael J Blanchette P Maleki Vareki S . Association between immune-related side effects and efficacy and benefit of immune checkpoint inhibitors – a systematic review and meta-analysis. Cancer Treat Rev. (2021) 92. doi: 10.1016/j.ctrv.2020.102134, PMID: 33302134

[2] Riley RS June CH Langer R Mitchell MJ . Delivery technologies for cancer immunotherapy. Nat Rev Drug Discov. (2019) 18:175–96. doi: 10.1038/s41573-018-0006-z, PMID: 30622344 PMC6410566

[3] Barcellini W Fattizzo B . The changing landscape of autoimmune hemolytic anemia. Front Immunol. (2020) 11:946. doi: 10.3389/fimmu.2020.00946, PMID: 32655543 PMC7325906

[4] Kroll MH Rojas-Hernandez C Yee C . Hematologic complications of immune checkpoint inhibitors. Blood. (2022) 139:3594–604. doi: 10.1182/blood.2020009016, PMID: 34610113 PMC9227102

[5] Yin Q Wu L Han L Zheng X Tong R Li L . Immune-related adverse events of immune checkpoint inhibitors: A review. Front Immunol. (2023) 14:1167975. doi: 10.3389/fimmu.2023.1167975, PMID: 37304306 PMC10247998

[6] Connolly C Bambhania K Naidoo J . Immune-related adverse events: A case-based approach. Front Oncol. (2019) 9:530. doi: 10.3389/fonc.2019.00530, PMID: 31293970 PMC6598598

[7] Scheckel CJ Go RS . Autoimmune hemolytic anemia: diagnosis and differential diagnosis. Hematol Oncol Clin North Am. (2022) 36:315–24. doi: 10.1016/j.hoc.2021.12.001, PMID: 35282951

[8] Barcellini W . New insights in the pathogenesis of autoimmune hemolytic anemia. Transfus Med Hemother. (2015) 42:287–93. doi: 10.1159/000439002, PMID: 26696796 PMC4678320

[9] Naimi A Mohammed RN Raji A Chupradit S Yumashev AV Suksatan W . Tumor immunotherapies by immune checkpoint inhibitors (Icis); the pros and cons. Cell Commun Signal. (2022) 20:44. doi: 10.1186/s12964-022-00854-y, PMID: 35392976 PMC8991803

[10] Wang SJ Dougan SK Dougan M . Immune mechanisms of toxicity from checkpoint inhibitors. Trends Cancer. (2023) 9:543–53. doi: 10.1016/j.trecan.2023.04.002, PMID: 37117135 PMC10330206

[11] Zhang L Mai W Jiang W Geng Q . Sintilimab: A promising anti-tumor pd-1 antibody. Front Oncol. (2020) 10:594558. doi: 10.3389/fonc.2020.594558, PMID: 33324564 PMC7726413

[12] Pang X Huang Z Zhong T Zhang P Wang ZM Xia M . Cadonilimab, a tetravalent pd-1/ctla-4 bispecific antibody with trans-binding and enhanced target binding avidity. mAbs. (2023) 15. doi: 10.1080/19420862.2023.2180794, PMID: 36872527 PMC10012886

[13] Frentzas S Austria Mislang AR Lemech C Nagrial A Underhill C Wang W . Phase 1a dose escalation study of ivonescimab (Ak112/smt112), an anti-pd-1/vegf-a bispecific antibody, in patients with advanced solid tumors. J ImmunoTherapy Cancer. (2024) 12. doi: 10.1136/jitc-2023-008037, PMID: 38642937 PMC11033648

[14] Don T Gadgeel M Savaşan S . A room for long-lived plasma cell contribution in immune cytopenias? Cancers. (2025) 17. doi: 10.3390/cancers17091537, PMID: 40361462 PMC12071925

[15] Adeoye FW Surandran S Jaffar N Serumadar S Begum G . Early-onset autoimmune hemolytic anemia from pembrolizumab in a patient with metastatic lung cancer: A case report. Am J Case Rep. (2025) 26:e946630. doi: 10.12659/AJCR.946630, PMID: 40121521 PMC11939122

[16] Smirnova SJ Sidorova JV Tsvetaeva NV Nikulina OF Biderman BV Nikulina EE . Expansion of cd8+ Cells in autoimmune hemolytic anemia. Autoimmunity. (2016) 49:147–54. doi: 10.3109/08916934.2016.1138219, PMID: 26829107

[17] de Boer ECW Mulder FVM Neri S Wiskerke-van Stuijvenberg M Arts JJG Tol S . Vesiculation as potential novel pathogenic mechanism in autoimmune hemolytic anemia. Transfusion. (2025) 65(6):1132–44. doi: 10.1111/trf.18270, PMID: 40351186 PMC12168431

[18] Arafat Hossain M . A comprehensive review of immune checkpoint inhibitors for cancer treatment. Int Immunopharmacol. (2024) 143. doi: 10.1016/j.intimp.2024.113365, PMID: 39447408

[19] Cook SL Al Amin M Bari S Poonnen PJ Khasraw M Johnson MO . Immune checkpoint inhibitors in geriatric oncology. Curr Oncol Rep. (2024) 26:562–72. doi: 10.1007/s11912-024-01528-3, PMID: 38587598

[20] Jacobs JW Raza S Clark LM Stephens LD Allen ES Woo JS . Mixed autoimmune hemolytic anemia: A systematic review of epidemiology, clinical characteristics, therapies, and outcomes. Am J Hematol. (2025) 100(8):1397–407. doi: 10.1002/ajh.27721, PMID: 40392014 PMC12232602

[21] Danziger M Hermantin I Valea F Tymon-Rosario J . Immune checkpoint inhibitor-induced autoimmune hemolytic anemia in endometrial cancer. Gynecologic Oncol Rep. (2025) 59. doi: 10.1016/j.gore.2025.101746, PMID: 40292083 PMC12033931

[22] Tao Y Li J Pan J Wang Q Ke RW Yuan D . Integration of scrna-seq and bulk rna-seq identifies circadian rhythm disruption-related genes associated with prognosis and drug resistance in colorectal cancer patients. Immunotargets Ther. (2025) 14:475–89. doi: 10.2147/ITT.S499806, PMID: 40241740 PMC12000913

