# Supplementary material for: Case Report: Challenges in immunotherapy for the elderly: a case of refractory ICI-induced AIHA and thrombocytopenia in advanced gastric cancer
Source: Front Immunol. 2025 Oct 22;16:1679817. doi: 10.3389/fimmu.2025.1679817 (PMC12586891; doi:10.3389/fimmu.2025.1679817)
Supplement: Supplementary file 1 [file DataSheet1.docx]

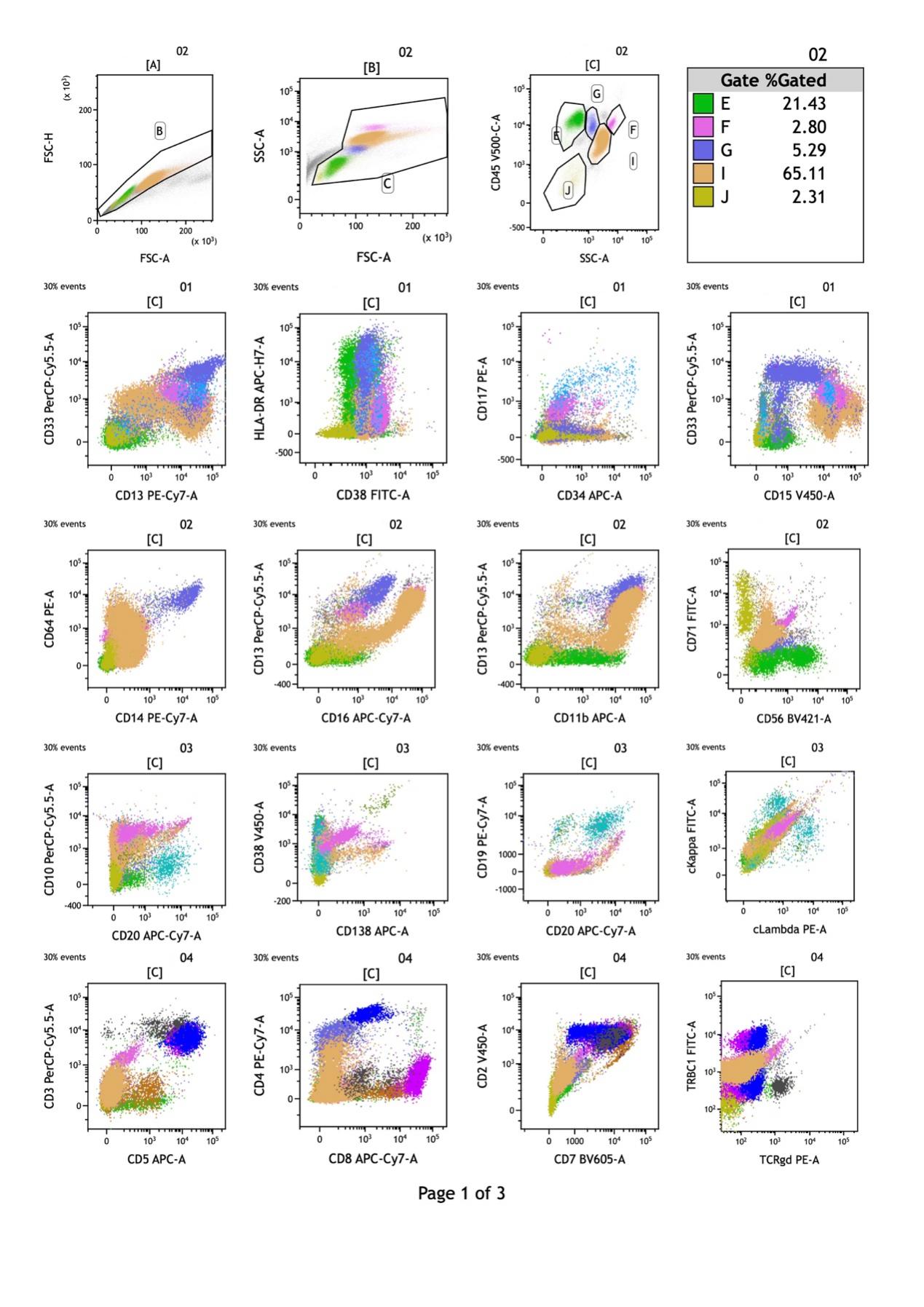


**FIGURE S1**

Multilineage hematopoietic populations displayed physiologically balanced distributions, with myeloid,T,B, and NK cell subsets exhibiting immunophenotypic profiles consistent with normal differentiation. No aberrant immunophenotypes or clonal expansions were detected, supporting an intact and functionally normal trilineage hematopoiesis and excluding early leukemic transformation.


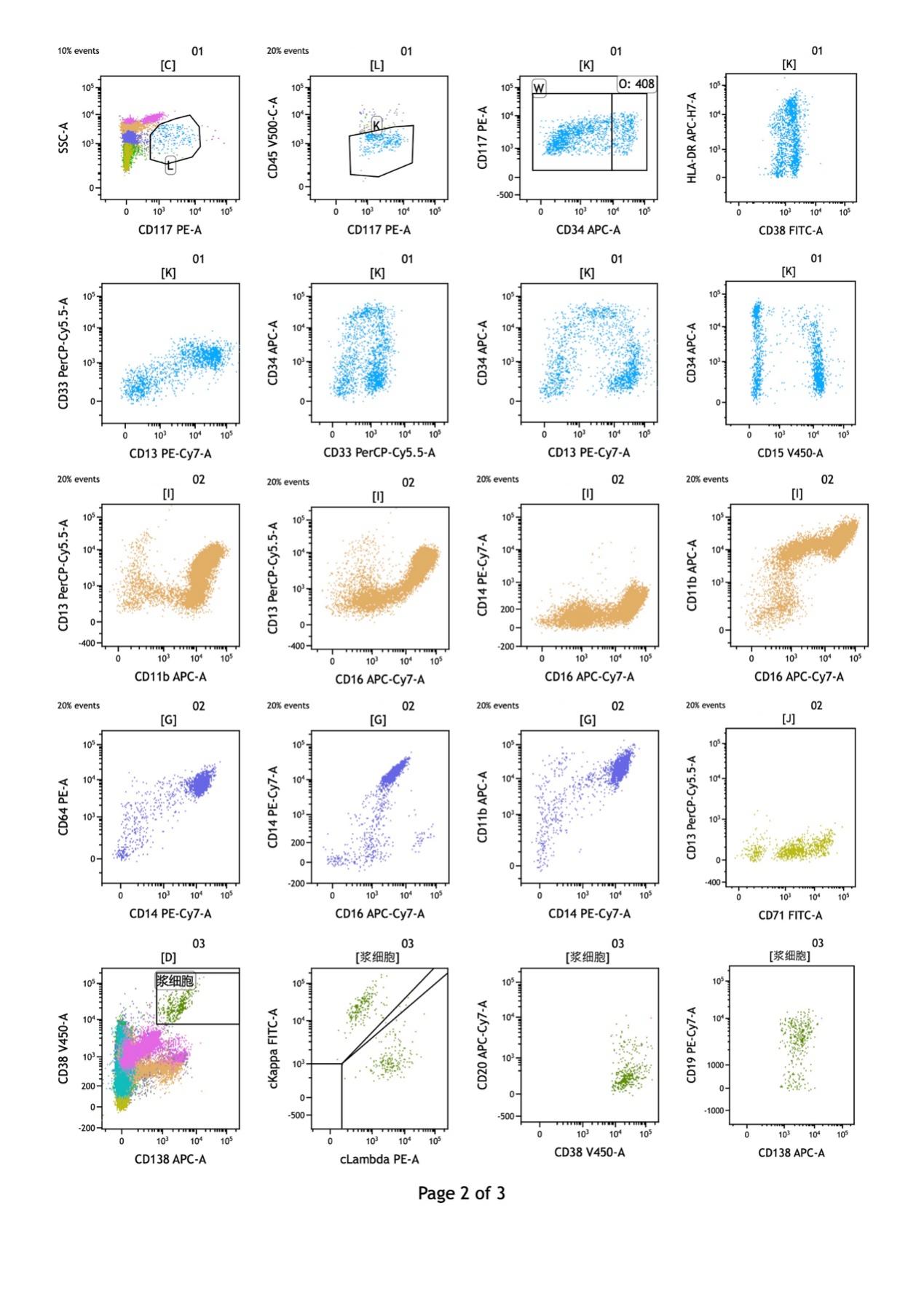


**FIGURE S2**

Myeloid progenitor cells expressing CD34 and CD117 exhibited normal immunophenotypic profiles, while mature myeloid and monocytic subsets defined by CD11b, CD13, CD14, and CD16 were uniformly distributed without evidence of aberrant expansion or maturation arrest, indicating intact and physiologically regulated myelopoiesis.


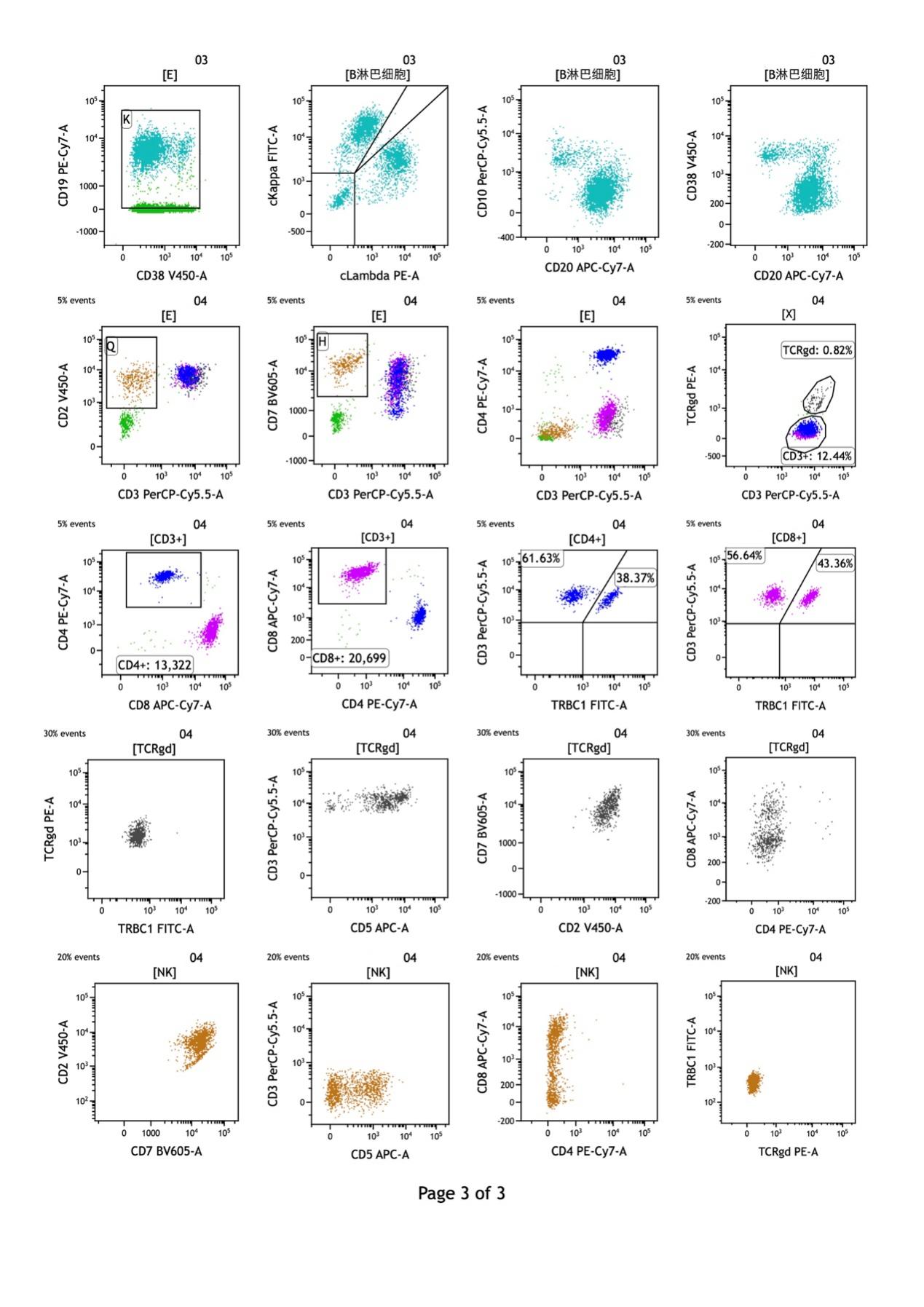


**FIGURE S3**

The distribution of T-cell subsets, including a physiologic CD4⁺ to CD8⁺ ratio, was within normal limits. TCRγδ⁺ T cells, B-cell light chain expression (κ/λ), and NK cell immunophenotypes were all consistent with non-clonal, antigenically diverse populations. No aberrant lymphoid expansions were observed, supporting an immunophenotypically intact and polyclonal lymphoid compartment.
